# Naturalist's Monthly Report

**Published:** 1811-08

**Authors:** 


### NATURALIST'S MONTHLY REPORT.

### JUNE.

FLOWERING MONTH.

> The martial pea observe,
> In square battalion rang'd, line after line,
> Successive; the gay bean, her hindmost ranks
> Stript of their blossoms; the thick-scatter'd bed
> Of soporific lettuce.

On the 1st and 2d of the month the wind was east in the morning, and westerly towards the latter part of the day; on the 3d westerly; on the 4th and 5th south-west; from the 6th to the 9th westerly; on the 10th south-west; on the 11th and 12th westerly; on the 13th and 14th south-west; on the 15th westerly; on the 16th south-west in the morning, and north-west in the evening; on the 17th easterly; on the 18th south; on the 19th easterly in the morning, and in the afternoon north; on the 20th and 21st easterly; on the 22d, 23d, and 24th, north-east; on the 25th, 26th, 27th, easterly; on the 28th south-west; and on the two last days of the month easterly.

There were strong gales on the 4th, 6th, 10th, and 12th, and fresh gales on the 8th and 9th. I do not recollect to have heard any thunder during this month. There were heavy showers in the morning of the 2d; and rain, more or less, on the 5th, 20th, 24th, 28th and 29th.

June 1st. The same singularity with respect to the tides, which was spoken of in all the public prints, occurred along the coast of Hampshire, this day, to the great astonishment of all who witnessed it. This phenomenon I have not yet (July 22d) seen accounted for.

June 3d. The bloom of the hawthorn is nearly all gone, having been in a great measure beaten off by the late heavy rains.

June 4th. The hay-harvest has commenced, but the weather is not very favourable.

The following wild herbaceous plants are now in flower:—Common buckbean *(menyanthes trifoliatum)*, mouse-ear scorpion-grass *(myosotis arvensis)*, yellow water lily *(nymphæa lutea)*, narrow-leaved pond-weed, *(polygonum amphibium)*, black bind-weed *(polygonum convolvulus)*, common broom rape *(orobanche major)*, and long-storked crane's-bill *(geranium columbinum.)*

June 7th. Mackarel have been caught in tolerable quantity along the coast. They are small, and are selling for ninepence per dozen.

June 8th. The pods of furze crack, and throw out their seeds. The stamina of the flowers of the nettle throw out their farina. They do this by a sudden expansion; and, in the sun-shine, the appearance is not unlike that of the explosion of so many grains of gun-powder.

June 9th. I this day saw a saffron-coloured butterfly on the wing, which most probably was the clouded yellow species *(papilio edusa* of Linnæus and Haworth), but its flight was so rapid that I could not perfectly distinguish it.

June 10th. The rivers are much discoloured by the rains which have fallen in the country to the westward and northward.

June 12th. The farmers are beginning to carry and stack their hay.

June 16th. Cherries are gathered. Wheat is in flower.

June 18th. The cuckoo begins to stammer.

In the evening of this day mackarel were again caught For several days past the shoals have kept at such a distance from the shore, that the seine nets of the fishermen could not reach them.

June 20th. Wheatears are in great numbers on the heaths.

June 22d. There was this morning a very sharp white frost.

June 24th. I remarked an immense number of swallows and martins flying about over a large field of pease. They were no doubt attracted to that particular spot by the insects that abounded there, of which they must have devoured myriads. The utility of these and other birds in thus checking the ravages of what is commonly termed blight, is incalculably great.

June 26th. The leaves of several kinds of forest trees, particularly the elms and limes, have been shrivelled up by the late cold winds, much in the manner that the foliage was two years ago, but by no means to the same extent.

July 28th. Some shoals of white mullet come into the harbour.

June 31st. The musk thistle *(carduus nutans)*, common tansy *(tanacetum vulgare)*, climbing fumitory *(fumaria claviculata)*, marsh St. John's wort *(hypericum elodes)*, common St. John's wort *(hypericum perforatum)*, ragwort *(senecio Jacobæa)*, greater daisy *(chrysanthemum leucanthemum)*, and corn marigold *(chrysanthemum segetum)* are in flower.

*Hampshire.*

METEORO-

